# Supplementary material for: Type 1 and type 2 diabetes mellitus: Clinical outcomes due to COVID-19. Protocol of a systematic literature review
Source: PLoS One. 2022 Sep 9;17(9):e0271851. doi: 10.1371/journal.pone.0271851 (PMC9462572; doi:10.1371/journal.pone.0271851)
Supplement: Attachment — Submitted filename: Response to Reviewers.docx [file pone.0271851.s003.docx]

**PONE-D-22-19262**

**Answers to reviewer # 1:**

**Comment#1.1:** Operational definition of COVID-19 Diagnosis is required.

**Response#1.1:** Thank you very much for your suggestion. The operational definition of COVID-19 used is established in the eligibility criteria section (participants or population). The correction is as follows:

*For the operational definition of confirmed cases of COVID-19, the guidelines established by the WHO will be used. A confirmed case of COVID-19 is defined as any positive SARS-CoV-2 nucleic acid amplification test or symptomatic person who finds a suspected or probable case plus positive SARS-CoV-2 antigen test or asymptomatic person with antigen test SARS-CoV-2 positive plus known contact of a probable or suspected case.*

**Comment#1.2:** Please provide diagnostic algorithm as per statement written.

**Response#1.2:** Thank you very much for your suggestion. The algorithm for diagnosis and definition of a confirmed case of COVID-19 used by the World Health Organization is expressed in the previous answer and the reference is provided in the manuscript.

**Comment#1.3:** Operational definition of diagnosis of diabetes mellitus is mandatory.

**Response#1.3:** Thank you very much for your suggestion. The operational definition of the diagnosis of diabetes mellitus is found in the section eligibility criteria (exposure). The correction is as follows:

*Patients with COVID-19 and concomitant diagnosis of unspecified diabetes mellitus, differentiated into type 1 diabetes mellitus or type 2 diabetes mellitus, without restriction of geographic region, gender, or age of the patients, who present definition of clinical criteria and /or paraclinical tests used by researchers to classify patients according to their diabetes status.*

*The operational definition of a confirmed case of diabetes mellitus provided by the American Diabetes Association will be used as a guide. The reference diagnostic criteria for diabetes are fasting plasma glucose ≥126 mg/dL (7.0 mmol/L). Fasting is defined as no caloric intake for at least 8 h or 2-h plasma glucose ≥ 200 mg/dL (11.1 mmol/L) during OGTT or hemoglobin A1C ≥6.5% (48 mmol/mol) or in a patient with classic symptoms of hyperglycemia or hyperglycemic crisis, at random plasma glucose ≥200 mg/dL.*

*In selected primary studies, identification of diabetes status may be based on medical history and International Classification of Diseases codes for type 1 or type 2 diabetes, use of antidiabetic medications, or previously defined diagnostic criteria.*

**Comment#1.4:** Please give heading of snowball manual search and provide details.

**Response#1.4:** Thank you very much for your suggestion. The correction is as follows and can be found in the information sources and search strategy section.

*To identify other potentially eligible studies, the references of relevant publications will be reviewed to perform a snowball manual search. This technique consists of searching for new articles from the primary studies already selected in order to guarantee exhaustiveness in the search.*

**Comment#1.5:** Identify limitation of study and given as separate heading.

**Response#1.5:** Thank you very much for your suggestion

*Limitations: there are some potential limitations to the proposed systematic review. Firstly, both type 1 and type 2 diabetes may have different key search terms and some studies may be missed. To minimize this limitation, different search equations have been designed for each database in an exhaustive and sensitive manner. In addition to reading references and level ball as an additional strategy. Another limitation is that observational studies evaluating the effect of an intervention may be susceptible to significant confounding bias and may present high heterogeneity in the findings. To report these possible biases, an adequate quality assessment will be carried out, with highly sensitive and previously validated tools, exclusive for each type of observational design. The review is intended for publication in a peer-reviewed journal.*

**Comment#1.6:** Another similar protocol found "Impact of virtual care on health-related quality of life in children with diabetes mellitus: a systematic review protocol" Justify your study and differentiate between previous studies and protocol.

**Response#1.6:** Thank you very much for your comment. Many systematic reviews and meta-analyses have been developed during the COVID-19 pandemic. Some of them with the aim of establishing the risk presented by patients with diabetes and COVID-19. However, the present research protocol allows for a comprehensive synthesis of the existing evidence related to the effects of COVID-19 in type 2 and type 1 diabetes compared to patients without diabetes. Until now, this synthesis of the literature is not available, and it is considered crucial to continue understanding the effects of the pandemic. In addition, our protocol proposes an ecological analysis where some interesting new hypotheses can be explored regarding this topic**.**

**Answers to reviewer # 2:**

Overall, this appears to be a well-written systematic review protocol, and the question seems to be worthwhile, with no previous systematic reviews to have comprehensively synthesized the existing evidence related to the effects of COVID-19 on type 2 and type 1 diabetes. The methods for article selection and extraction appear generally sound. The quality of this protocol could be improved by considering the following points.

**Comment#2.1:** Line 96: delete the word “design”.

**Response#2.1:** Thank you very much for your suggestion. We remove the word “design” from line 96.

**Comment#2.2:** Line 132: it is not clear how the authors will treat studies that had COVID-19 and diabetes co-morbidities but without the outcomes of interest for this systematic review.

**Response#2.2:** Thank you very much for your suggestion. Only articles whose main hypothesis is to assess the effect of diabetes (type 1 or type 2) on the three established outcomes of COVID-19 will be included in the systematic review. It is explained in the exclusion criteria section.

*Clinical trials, editorials, letters to the editor, reviews, case reports, case series, narrative reviews or systematic reviews and meta-analyses, as well as research in the field of basic sciences based on experimental laboratory models, will be excluded. Original research articles that only include other types of diabetes, such as monogenic diabetes, gestational diabetes, latent autoimmune diabetes in adults, ketosis-prone diabetes, among others, or articles with publication status prior to publication will not be considered. revision. In addition, articles whose main hypothesis is not diabetes and do not have the established outcomes will be excluded.*

**Comment#2.3:** Authors could consider to search additional databases such as SCOPUS, CINAHL, Web of Science to ensure all potential studies are captured.

**Response#2.3:** Thank you very much for your suggestion. We consider including the SCOPUS database in the search strategy in order to guarantee exhaustiveness in the systematic review.

*The following electronic bibliographic databases will be used: MEDLINE, EMBASE, LILACS, OVID MEDLINE, WHO (COVID-19 Global literature on coronavirus disease) and SCOPUS with a publication date from December 2019 to March 20, 2022, without language restriction.*

**Comment#2.4:** It was not clear how the authors will treat gray literature, since these often contain useful data that could enhance the quality of systematic review.

**Response#2.4:** Thank you very much for your suggestion. The gray literature search for the systematic review will be carried out in the WHO database (COVID-19 Global literature on coronavirus disease). This database contains the gray literature on the subject of COVID-19. The correction is as follows:

*The search for potential primary studies published in gray literature will be performed through the World Health Organization database for COVID-19 (WHO COVID-19 Global literature on coronavirus disease). This database contains different electronic bibliographic databases incorporated into its browser, including Web of Science, EuropePMC and Gray literature, among others.*

**Comment#2.5:** Line 155-156: how discrepancies between data extractors will be resolved? Authors could choose one method and consistently apply it rather than providing options that may risk reproducibility of the review.

**Response#2.5:** Thank you very much for your comment. The text has been organized in a clearer way. It is located in the Study selection process section.

*Two researchers will independently evaluate all the titles and abstracts of the retrieved articles, using the free access Rayyan® software [14] with previously established selection criteria. Disagreements will be resolved in the first instance through discussion and in the second instance through a third reviewer. After the articles selected in the eligibility phase, the full text will be read independently by two researchers, both using the same instrument previously validated in Excel according to predefined criteria. Discrepancies will be resolved by discussion or a third reviewer. The process of identification, selection and inclusion of primary studies will be described and presented using the flowchart recommended by the PRISMA statement in its latest version 2020.*

**Comment#2.6:** I'm also curious to know what the authors plan to do if they identify a study without its full-text pdf. Also, I assume the authors will be extracting data from full-text articles, but this could be clearer.

**Response#2.6:** Thank you very much for your comment. The text has been organized in a clearer way. It is located in the Data collection and extraction section.

*Standardized and validated forms will be used to collect the data extracted from the primary studies, accompanied by a detailed instruction manual to specify the guiding questions, and avoid the introduction of bias. Data will be extracted from those articles in full text format. If the full text is not available, contact the author or search for the manuscript with the help of the library system. This process will be carried out by two researchers independently. A third investigator will verify the extracted data to ensure the accuracy of the records. The authors of the primary studies will be contacted to resolve any questions that may arise. The reviewers will resolve the disagreements through discussion and one of the two referees will adjudicate the discrepancies presented through discussion and consensus.*
